# Association of Grandparental and Parental Age at Childbirth With Autism Spectrum Disorder in Children

**DOI:** 10.1001/jamanetworkopen.2020.2868

**Published:** 2020-04-15

**Authors:** Yu Gao, Yongfu Yu, Jingyuan Xiao, Jiajun Luo, Yawei Zhang, Ying Tian, Jun Zhang, Jørn Olsen, Jiong Li, Zeyan Liew

**Affiliations:** 1 Department of Environmental Health, School of Public Health, Shanghai Jiao Tong University School of Medicine, Shanghai, China; 2 Department of Clinical Epidemiology, Aarhus University Hospital, Aarhus, Denmark; 3 Department of Environmental Health Sciences, Yale School of Public Health, New Haven, Connecticut; 4 Yale Center for Perinatal, Pediatric, and Environmental Epidemiology, Yale School of Public Health, New Haven, Connecticut; 5 The Ministry of Education–Shanghai Key Laboratory of Children’s Environmental Health, Xinhua Hospital, Shanghai Jiao Tong University School of Medicine, Shanghai, China

## Abstract

**Importance:**

Advanced parental age has been associated with autism spectrum disorders (ASDs) in children. However, little is known about the association between grandparental age at the time of birth of the parent and the risk of ASD in the grandchildren.

**Objective:**

To estimate the associations between parental and grandparental age and ASD risk in children.

**Design, Setting, and Participants:**

This population-based, multigenerational cohort study used data from Danish national health registries. A parental age cohort was constructed to evaluate the association between parental age and ASD in 1 476 783 singleton children born from 1990 to 2013, and a multigenerational cohort was also constructed including 362 438 fathers and 458 234 mothers born from 1973 to 1990 for whom information on grandparental age was available. Data analyses were conducted from November 1, 2018, through February 7, 2020.

**Exposures:**

Parental age at childbirth and grandparental age at the time of the birth of the parent.

**Main Outcomes and Measures:**

Diagnoses of ASD in children were obtained from the Danish Psychiatric Central Register (1994-2017). Logistic regression analysis was used to estimate the associations between parental or grandparental age and ASD in children.

**Results:**

Of the 1 476 783 children born from 1990 to 2013, 758 066 (51.3%) were male, and 27 616 (1.9%) had ASD (20 467 [74.1%] were male). Advanced paternal or maternal age over 30 years was monotonically associated with increased ASD risk, with odds ratios (ORs) of 1.56 (95% CI, 1.45-1.68) for maternal age 40 years and older and 1.57 (95% CI, 1.39-1.78) for paternal age 50 years and older, compared with parents aged 25 to 29 years. In the multigenerational cohort, 9364 grandchildren (1.7%) had ASD. This study found U-shaped associations, in that ASD risk was higher among grandchildren of younger (≤19 years) maternal grandmothers (OR, 1.68; 95% CI, 1.52-1.85), younger maternal grandfathers (OR, 1.50; 95% CI, 1.26-1.78), and younger paternal grandmothers (OR, 1.18; 95% CI, 1.04-1.34), and older (≥40 years) paternal grandmothers (OR, 1.40; 95% CI, 1.03-1.90) compared with the grandchildren of grandparents who were aged 25 to 29 years at the time of giving birth to the parents.

**Conclusions and Relevance:**

These findings corroborate previous studies suggesting that advanced parental age is independently associated with increased ASD risk in children. This study also found that children with young maternal grandparents and children with young and old paternal grandparents had elevated ASD risk. Possible transmission of ASD risk across generations should be considered in etiological research on ASD.

## Key Points

**Question:**

Is grandparental age at the time of birth of the parent associated with the risk for autism spectrum disorders (ASDs) in the grandchildren?

**Findings:**

This Danish national population-based cohort study across 3 generations observed transgenerational associations suggesting that ASD risk in children was elevated if their mothers were born to young (≤19 years) grandparents or if their fathers were born to young (≤19 years) and older (≥40 years) grandparents, compared with children whose parents were born to grandparents aged 25 to 29 years. These associations observed for grandparental hage were independent of possible parental age associations with ASD risk in children.

**Meaning:**

These findings suggest that the risk of ASD associated with young or advanced grandparental age might be transmitted across generations, which should be considered in future research of the causes of ASD.

## Teaser

This cohort study estimates the associations between grandparental age at the time their children were born and the risk of autism spectrum disorders in their grandchildren.

## Introduction

Autism spectrum disorder (ASD) is a neurodevelopmental disorder characterized by communication deficiencies, language impairments, and repetitive patterns of behavior.^1^ The prevalence of ASD among children has been increasing during the past decades, and the prevalence is estimated to be approximately 1% to 2% across different regions.^2^ In the US, the Centers for Disease Control and Prevention reported that children born in 2006 had a more than 2-fold risk of ASD compared with those born a decade previously.^3^ This increase in prevalence may be attributed to a combination of factors, such as improved diagnostic efficiency and heightened awareness and/or a true increase.^4^ Although etiological research in ASD has focused predominantly on genetic factors, perinatal and environmental risk factors likely contribute to the increased ASD prevalence over time as well.^5,6^

In light of worldwide increasing trends regarding postponed parenthood, the possible association of parental age on child health has generated considerable interest.^7^ Research conducted in different study populations has suggested that advanced maternal and paternal age are independently associated with increased risk of ASD in children,^8,9,10,11^ for which different mechanisms have been proposed.^12^ Increased rates of de novo mutations and epigenetic alternations associated with increasing age are the most frequently cited mechanisms to explain the association between paternal age and ASD risk in children.^13,14^ Meanwhile, higher rates of chromosomal abnormalities, perinatal and obstetric complications, and potential genomic and/or epigenetic alterations induced by cumulative exposure to environmental toxins might account for the association between maternal age and ASD risk in children.^15^

More recently, hypotheses regarding the possibility of transgenerational exposure risk for ASD have been raised. An increasing number of animal experiments^16,17^ have indicated that intrauterine environmental exposures could lead to de novo and/or epigenetic alterations in the germline that could subsequently influence disease risk in future generations. Although most of these findings have come from animal studies,^16,17,18^ epidemiological evidence of transgenerational exposure effects on neurological diseases risk is also emerging.^19,20,21,22^ A UK birth cohort study^19^ found that grandmaternal smoking has been linked to ASD risk in granddaughters. In addition, grandmaternal intake of diethylstilbestrol during pregnancy has been associated with attention-deficit/hyperactivity disorder diagnosis in the grandchildren, as reported in a US cohort.^20^ Advanced grandfather’s age was also found to be associated with increased risk for ASD and schizophrenia in 2 Swedish studies.^21,22^ We hypothesized that grandparental age at the time of birth of the parents might affect de novo and/or epigenetic alterations in the germline of the parents and subsequently be associated with ASD risk in the grandchildren. We conducted a population-based multigenerational cohort study and investigated whether ASD risk in children was associated with parental age at childbirth and also with grandparental age at the time of birth of the parents.

## Methods

### Study Population

The study was approved by the Danish Data Protection Agency and the institutional review board at Yale University. Informed consent was not required according to Danish law governing registry-based research studies with no participant contact.

**Figure 1.**
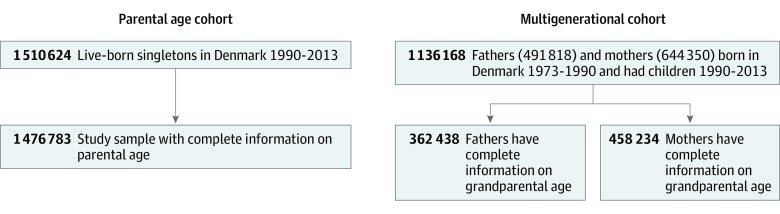
Flowchart of Parental Age Cohort and Multigenerational Cohort Sample Selections

The unique personal identification number assigned to all Danish residents allows the linkage across registers, including the Civil Registration System (CRS)^23^ and the Danish Medical Birth Register (DMBR).^24^ We constructed a parental age cohort by including 1 476 783 singleton children born from 1990 to 2013 in Denmark. The parental age cohort was constructed to investigate the association between parental age at delivery and ASD risk in children. The study period was selected to allow appropriate follow-up for ASD ascertainment using the Danish Psychiatric Central Register. We also constructed a multigenerational cohort by including all fathers and mothers born in Denmark from 1973 to 1990 to study the association between grandparental age and ASD risk in the grandchildren born from 1990 to 2013. A total of 362 438 and 458 234 singleton children had paternal or maternal grandparental age information, respectively, available for analyses. Children were followed from birth until December 31, 2017, for ASD status. The flowchart of the study participants’ selection is presented in Figure 1.

### ASD Diagnoses

Diagnoses of ASD were ascertained from the DPCR^25^ using the *International Statistical Classification of Diseases and Related Health Problems, Tenth Revision *(*ICD-10*), which has been used from 1994 to the present.^26^ Child psychiatrists who had mandatory training in the use of the *ICD-10* were responsible for registry reporting.^23^ Autism spectrum disorders were typically diagnosed at ages 3 to 5 years.^27^ Children born in 1990 were approximately 4 years old in 1994, and children born in 2013 would be at least 3 years of age by the end of study follow-up in 2017. A child was considered to have ASD if he or she had received a diagnosis with at least 1 of the following *ICD-10* codes: F84.0, F84.1, F84.5, F84.8, and F84.9.

### Statistical Analysis

We used logistic regression to calculate odds ratios (ORs) and 95% CIs for ASD risk in children and evaluated the associations of maternal age, paternal age, maternal grandparental age, and paternal grandparental age with ASD risk in children separately. Parental age was defined as the parent’s age at the time of the index child’s birth. Grandparental age was defined as the grandparent’s age at the time of birth of the parent. We first analyzed parental age and grandparental age as continuous variables and estimated the risk for ASD associated with a 5-year increase in age. An age square term was included in the model to evaluate for potential nonlinear response for continuous age variables and ASD risk. To facilitate comparisons with previous studies,^8,10,21^ age variables were grouped into 5-year intervals and categorized into the following 6 categories: younger than 20 years, 20 to 24 years, 25 to 29 years, 30 to 34 years, 35 to 39 years, and 40 years or older. The oldest age group was further classified into 40 to 49 years and 50 years or older for paternal age, and 45 years or older for grandpaternal age. The reference group was set to 25 to 29 years for categorical age analyses.

Potential confounders were selected a priori according to directed acyclic graphs (see eFigure 1 and eFigure 2 in the Supplement). The main covariate data included parity, the date of birth, and child’s sex retrieved from the DMBR, and parental age and maternal country of origin obtained from the CRS. Information on parental education was linked from the Integrated Database for Labour Market Research.^28^ In the analyses for parental age, we adjusted for child’s birth year, parity of the mother, maternal education, and maternal country of origin. In the analyses for grandparental age, we adjusted for birth year of the parents, parity of the grandmothers at the time of the parent’s birth, and grandmaternal education. We constructed a separate model that also adjusted for the age of the spouse in the analyses to disentangle the potential independent effect of age from 1 parent. However, spouse age was moderately to highly correlated in our data (Pearson correlation coefficients of 0.67 for parental age and 0.73 for grandparental age); thus, collinearity is possible, and decreased statistical precision are expected in the model after mutually adjusting for the spouse’s age.

Genetic confounding by mental illnesses might influence the association between parental age of delivery and ASD risk in children^29^; thus, we also performed sensitivity analyses to also adjust for the psychiatric history (*ICD-10* codes F00-F99) of either parent before the child’s birth in the analyses for parental age. Because the information on *ICD-10* diagnoses became available from 1994, we restricted the analyses with additional adjustment for parental psychiatric illnesses to parents who gave birth between 2000 and 2013, allowing at least 5 years for disease diagnosis before giving birth. Psychiatric history for grandparents before the birth of the parents were not available in the *ICD-10* records, and only 4% of grandmothers had a country of origin other than Denmark; therefore, these covariates were not included for grandparental age analyses. In addition, because paternal education information was obtained for only 95% of fathers (5% missing) from the Integrated Database for Labour Market Research, we presented the results of paternal education adjustment as a sensitivity analysis. However, grandparental education is largely missing, and we were, thus, unable to adjust for grandparental education in grandpaternal age analyses. Furthermore, we performed stratified analyses and evaluated the potential effect measure modification by child’s sex in both parental and grandparental age analyses. We also evaluated the association of parental or grandparental age with specific ASD subtypes (eg, childhood autism [*ICD-10* code F84.0], Asperger syndrome [*ICD-10* code F84.5], or others) and performed analyses restricted to first-born children or first-born parents only.

Parental birth records registered in the DMBR since 1973 were available for only a subset of children (37.3%) born in 1990 to 2013 who were included in both study cohorts. Although the DMBR started in 1973, the CRS had parental age at birth information recorded starting from 1942. We conducted additional analyses by extending the multigenerational cohort to include fathers and mothers born in Denmark from 1942 to 1990 and reanalyzed the association between grandparental age and ASD in grandchildren. The increased sample size allowed us to further classify the oldest grandparental age group into 40 to 49 years and 50 years or more, which is comparable to parental age classification. Moreover, with an extended follow-up period, there was a sufficiently large number of children (90.3%) born from 1990 to 2013 who had both parental and grandparental age available, allowing us to evaluate whether the associations for grandparental age and ASD risk in grandchildren would change when parental age was also adjusted in the model. However, the birth year of the parents was the only covariate included in these analyses because variables such as grandmaternal education and parity obtained through DMBR were missing before 1973. All analyses were performed in SAS statistical software version 9.4 (SAS Institute). Data analyses were conducted from November 1, 2018, through February 7, 2020.

## Results

### Demographic Characteristics of the Study Population

Of the 1 476 783 children (758 066 [51.3%] male) born from 1990 to 2013 included in the parental age cohort, 27 616 (1.9%) had ASD (20 467 [74.1%] male); 9364 grandchildren (1.7%) in the multigenerational cohort had ASD. Most of the mothers were from Denmark (1 286 903 children [87.1%]) and most mothers had an upper secondary-level education or a bachelor’s degree or higher (1 135 683 children [76.9%]) (Table 1). The mean (SD) age at delivery was higher for the fathers compared with the mothers (32.5 [5.7] years vs 29.8 [4.9] years), and the age of delivery had increased considerably in similar rates for the fathers and mothers from 1990 to 2013 (eFigure 3 in the Supplement).

**Table 1.** Characteristics of the Study Participants in the Parental Age Cohort in Denmark

Characteristic	Parental age cohort (N = 1 476 783)^a^
Sex of children	
Male	758 066 (51.3)
Female	718 593 (48.7)
Missing	124 (0.01)
Birth year of children	
1990-1994	311 116 (21.1)
1995-1999	322 993 (21.9)
2000-2004	310 903 (21.0)
2005-2009	306 592 (20.7)
2010-2013	225 179 (15.3)
Parity of the mother	
1	647 801 (43.9)
2	550 614 (37.3)
≥3	278 368 (18.8)
Maternal education	
Primary and low secondary	315 194 (21.3)
Upper secondary and academy profession degree	684 001 (46.3)
Bachelor’s degree or higher	451 682 (30.6)
Missing	25 906 (1.8)
Maternal country of origin	
Country other than Denmark	188 930 (12.8)
Denmark	1 286 903 (87.1)
Missing	950 (0.1)

^a^ Singleton births in 1990 to 2013 with complete information on parental age in Demark.

Among grandmothers, nearly all were from Denmark (783 665 women [95.5%]), and more than one-half (439 460 women [53.5%]) had an upper secondary-level education or a bachelor’s degree (Table 2). The mean (SD) age at delivery for the grandparents (maternal grandfathers, 28.7 [5.3] years; maternal grandmothers, 25.9 [4.6] years; paternal grandfathers, 28.7 [5.2] years; paternal grandmothers, 25.9 [4.6] years) was lower than those of the parents, which remained rather stable over time from 1973 to 1990 (eFigure 3 in the Supplement).

**Table 2.** Characteristics of the Study Participants in the Multigenerational Cohort in Denmark^a^

Characteristics	Fathers (n = 362 438)	Mothers (n = 458 234)
Birth year of the parents		
1973-1977	196 262 (54.2)	225 543 (49.2)
1978-1982	116 352 (32.1)	150 334 (32.8)
1983-1987	42 425 (11.7)	67 433 (14.7)
1988-1990	7399 (2.0)	14 924 (3.3)
Grandmaternal parity at the time giving birth to the parents		
1	147 134 (40.6)	190 424 (41.5)
2	141 980 (39.2)	175 328 (38.3)
≥3	73 324 (20.2)	92 482 (20.2)
Grandmaternal education at the time giving birth to the parents		
Primary and low secondary	160 476 (44.3)	212 267 (46.3)
Upper secondary and academy profession degree	137 568 (37.9)	169 507 (37.0)
Bachelor’s degree or higher	60 801 (16.8)	71 584 (15.6)
Missing	3593 (1.0)	4876 (1.1)
Grandmaternal country of origin		
Country other than Denmark	14 629 (4.0)	18 259 (4.0)
Denmark	345 931 (95.5)	437 734 (95.5)
Missing	1878 (0.5)	2241 (0.5)

^a^ Fathers and mothers born in 1973 to 1990 (with complete information on grandparental age information recorded at birth) and had given birth during 1990 to 2013.

### Parental Age and Autism

When parental age was analyzed continuously, we estimated that a 5-year increase in maternal or paternal age was associated with a 9% increase in odds for ASD in children, whereas the effect size attenuated to a 3% increase in odds for maternal age (OR, 1.03; 95% CI, 1.02-1.05) and 7% (OR, 1.07; 95% CI, 1.06-1.09) for paternal age after mutually adjusting for the spouse’s age (eTable 1 in the Supplement).

**Figure 2.**
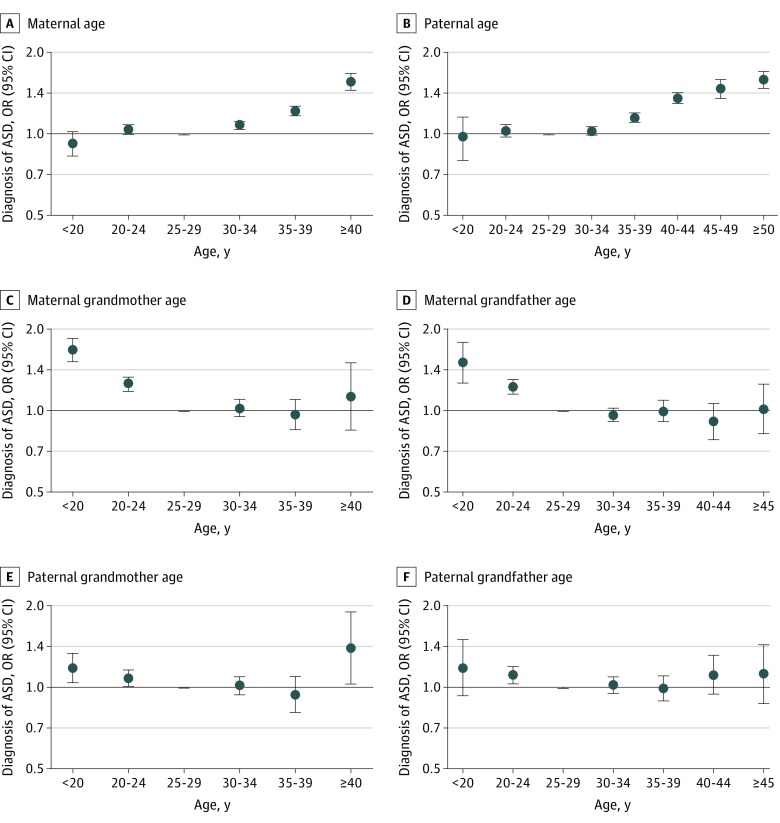
Odds Ratios (ORs) and 95% Confidence Intervals for Diagnosis of Autism Spectrum Disorder (ASD) in Children According to Parental and Grandparental Age (Years) at Delivery Dots denote ORs and error bars denote 95% CIs.

In 5-year categorization of age, the proportion of ASD among children born to mothers or fathers at ages 25 to 30 years was 1.84% and 1.90%, respectively. Maternal and paternal age of 30 years or greater was associated with a monotonic increase in ASD risk in children (Table 3 and Figure 2A and B). The highest OR was observed for maternal age over 40 years (OR, 1.56; 95% CI, 1.45-1.68), and paternal age greater than 50 years (OR, 1.57; 95% CI, 1.39-1.78) compared with ages 25 to 29 years as the reference. Few parents gave birth at ages younger than 19 years in this cohort, whereas maternal age of 20 to 24 years also was associated with a small increased risk for ASD (OR, 1.04; 95% CI, 1.00-1.08) compared with the reference. These effect estimates were slightly attenuated when spouse age was mutually adjusted in the model, but the overall associations remained (Table 3). The results did not change when we further adjusted for psychiatric history of the parents (eTable 2 in the Supplement). The association of paternal age with ASD in children was only slightly attenuated after additionally controlling for paternal education and the overall findings remain the same (eTable 3 in the Supplement).

**Table 3.** Diagnosis of ASD in Children According to Parental and Grandparental Age (Years) at Delivery

Variable	Children with ASD, No.		OR (95% CI)	
	Yes	No	Model 1	Model 2
Maternal age, y^a^				
≤19	419	19 005	0.92 (0.83-1.02)	0.94 (0.84-1.05)
20-24	3893	176 842	1.04 (1.00-1.08)	1.05 (1.01-1.10)
25-29	9353	498 454	1 [Reference]	1 [Reference]
30-34	9155	507 045	1.08 (1.04-1.11)	1.04 (1.00-1.07)
35-39	3960	211 177	1.22 (1.17-1.27)	1.10 (1.05-1.15)
≥40	836	36 644	1.56 (1.45-1.68)	1.31 (1.21-1.42)
Paternal age, y^a^				
≤19	120	5093	0.96 (0.80-1.15)	1.00 (0.81-1.20)
20-24	1886	85 626	1.02 (0.97-1.08)	1.01 (0.95-1.07)
25-29	7028	362 535	1 [Reference]	1 [Reference]
30-34	9464	526 094	1.02 (0.99-1.06)	1.02 (0.98-1.05)
35-39	5838	316 467	1.14 (1.10-1.19)	1.11 (1.07-1.16)
40-44	2322	111 036	1.35 (1.29-1.42)	1.27 (1.20-1.34)
45-49	693	31 262	1.46 (1.35-1.58)	1.35 (1.24-1.47)
≥50	265	11 054	1.57 (1.39-1.78)	1.44 (1.27-1.64)
Maternal grandmother age, y^b^				
≤19	601	23 418	1.68 (1.52-1.85)	1.56 (1.39-1.74)
20-24	2957	157 907	1.25 (1.18-1.33)	1.22 (1.15-1.30)
25-29	2681	176 418	1 [Reference]	1 [Reference]
30-34	1168	72 521	1.02 (0.95-1.10)	1.01 (0.93-1.09)
35-39	279	17 647	0.97 (0.85-1.10)	0.96 (0.83-1.10)
≥40	51	2586	1.13 (0.85-1.50)	1.12 (0.83-1.52)
Maternal grandfather age, y^b^				
≤19	141	5578	1.50 (1.26-1.78)	1.20 (1.00-1.45)
20-24	1800	88 583	1.22 (1.15-1.30)	1.11 (1.04-1.18)
25-29	3055	186 817	1 [Reference]	1 [Reference]
30-34	1813	114 277	0.96 (0.91-1.02)	1.01 (0.94-1.08)
35-39	654	38 889	0.99 (0.91-1.09)	1.05 (0.95-1.16)
40-44	184	11 451	0.91 (0.78-1.06)	0.97 (0.82-1.14)
≥45	90	4902	1.01 (0.82-1.25)	1.07 (0.85-1.34)
Paternal grandmother age, y^c^				
≤19	328	17 246	1.18 (1.04-1.34)	1.13 (0.98-1.30)
20-24	2012	121 846	1.08 (1.01-1.16)	1.07 (0.99-1.15)
25-29	2098	142 482	1 [Reference]	1 [Reference]
30-34	889	59 116	1.02 (0.94-1.10)	1.00 (0.92-1.10)
35-39	204	14 186	0.94 (0.81-1.10)	0.91 (0.77-1.07)
≥40	44	1987	1.40 (1.03-1.90)	1.30 (0.93-1.80)
Paternal grandfather age, y^c^				
≤19	73	3811	1.18 (0.93-1.50)	1.11 (0.86-1.43)
20-24	1185	68 816	1.11 (1.03-1.19)	1.07 (0.99-1.16)
25-29	2248	149 105	1 [Reference]	1 [Reference]
30-34	1385	91 618	1.02 (0.95-1.09)	1.03 (0.96-1.11)
35-39	459	30 769	0.99 (0.89-1.10)	1.02 (0.91-1.15)
40-44	161	9209	1.11 (0.94-1.31)	1.14 (0.95-1.37)
≥45	64	3535	1.12 (0.87-1.44)	1.11 (0.84-1.46)

Abbreviations: ASD, autism spectrum disorder; OR, odds ratio.

^a^ Model 1 was adjusted for child birth year, maternal parity, maternal education, and maternal country of origin. Model 2 was adjusted for child birth year, maternal parity, maternal education, maternal country of origin, and age of parent’s spouse.

^b^ Model 1 was adjusted for maternal birth year, parity of maternal grandmother, and education of maternal grandmother. Model 2 was adjusted for maternal birth year, parity of maternal grandmother, education of maternal grandmother, and age of maternal grandparent’s spouse.

^c^ Model 1 was adjusted for paternal birth year, parity of paternal grandmother, and education of paternal grandmother. Model 2 was adjusted for paternal birth year, parity of paternal grandmother, education of paternal grandmother, and age of paternal grandparent’s spouse.

### Grandparental Age and ASD

The associations for grandparental age and ASD in the grandchildren followed a nonlinear pattern (eTable 4 in the Supplement). In 5-year categorization of age, the proportion of ASD among children born to maternal grandmother, maternal grandfather, paternal grandmother, and paternal grandfather at ages 25 to 30 years was 1.50%, 1.61%, 1.45%, and 1.49%, respectively. We observed U-shaped associations between paternal grandparental age and ASD risk in the grandchildren by the 5-year groups—that is, the risk of ASD in grandchildren was increased for those whose fathers were born to young (aged ≤19 years) grandmothers (OR, 1.18; 95% CI, 1.04-1.34) and grandfathers (OR, 1.18; 95% CI, 0.93-1.50) or older (aged ≥40 years) grandmothers (OR, 1.40; 95% CI, 1.03-1.90) and grandfathers (OR, 1.11; 95% CI, 0.94-1.31) compared with grandparents aged 25 to 29 years as the reference. Moreover, children of mothers born to young (aged ≤19 years) grandmothers (OR, 1.68; 95% CI, 1.52-1.85) and grandfathers (OR, 1.50; 95% CI, 1.26-1.78) also had elevated risk of ASD, but no apparent associations were observed for older maternal grandparents (Table 3 and Figure 2C-F). Mutual adjustment for spouse’s age attenuated some of the associations toward the null, especially for younger grandpaternal age, but the overall associations remained (Table 3).

### Supplementary Analyses

There were no clear differences in the associations stratified by ASD subtypes, especially for the results regarding childhood autism and Asperger syndrome (eTable 5 in the Supplement). We did not find apparent differences by child’s sex either (eTable 6 in the Supplement). Results remained robust when we restricted the parental age analyses to first-born children, and also when we restricted the grandparental age analyses to first-born parents (eTable 7 in the Supplement).

When expanding birth year of the parents from 1942 to 1990 in the multigenerational cohort, attenuations of the younger grandparental age effect estimates and ASD risk in the grandchildren were noted, whereas the older maternal and paternal grandfather age effect estimates were strengthened (eTable 8 in the Supplement). Advanced paternal grandfather age effect estimates were slightly increased when spouse’s age was included in the model. The overall results for grandparental age and ASD risk in grandchildren were robust and only slightly attenuated when parental age was adjusted in the model (eTable 8 in the Supplement).

## Discussion

In this large, national, population-based cohort study across 3 generations, we found that both parental and grandparental ages at the time of birth of the children or the corresponding parents were associated with ASD risk in children. The associations between grandparental age and ASD risk in grandchildren persisted after adjusting for parental age.

Advanced parental age has already been considered as a potential risk factor for ASD.^8,29,30^ Our findings are consistent with those of previous large population-based studies,^9,10,31,32^ which indicated that both advanced maternal and paternal age were independently associated with increased risk of ASD in children. A newer hypothesis has recently been proposed that the association of parental age with the risk of ASD could persist across generations.^29^ One animal study^17^ has provided evidence that both the offspring and the second-generation offspring from older dams had altered sociability, grooming, and anxiety behaviors that were associated with the core symptoms of ASD. However, evidence from human studies is still lacking. Only 2 epidemiological studies^21,33^ have been conducted to investigate the association between grandparental age and ASD risk in grandchildren. The larger nested case-control study^21^ conducted in Sweden included 5936 individuals with childhood autism and reported that advanced grandpaternal age at the time of birth of the parent was associated with autism in grandchildren. However, the other smaller study^33^ from the UK with 86 ASD cases found that advanced maternal grandmother age was most significantly associated with ASD and autistic traits in the grandchildren. In our study, we did not find evidence to suggest that advanced maternal grandmother age was associated with ASD risk in grandchildren. Although we also observed an association of advanced paternal grandparental age and ASD risk in grandchildren, the estimated magnitude of effect is smaller than the reports from the Swedish study.^21^ However, we unexpectedly observed that the ASD risk in grandchildren was elevated for young grandparents in Denmark, especially among those who gave birth to parents before age 20 years.

Age at delivery is the result of diverse social and biological processes and might be a proxy for multiple risk factors that converge on ASD.^29^ Paternal age–related de novo mutations are widely assumed to be the underlying causal mechanism to neurodevelopmental disorders, including ASD and schizophrenia.^21,22,34^ However, genetic confounding has been raised as another plausible alternative explanation,^35,36^ as supported by a study^37^ reporting that women with a high genetic predisposition to schizophrenia also tended to have their first child at an early or later age. It is worth noting that the potential causes of delayed reproductive age might also influence ASD risk. Advanced age is associated with gamete dysfunction and fertility problems, which will delay reproduction.^38^ In addition, individuals with genetic liabilities may exhibit personality traits such as aloofness, rigidity, and anxiousness that would have adverse effects on their socioeconomic achievement, thereby delaying reproduction to an older age.^29,39^

The literature concerning mental health outcomes in young parenthood has been overshadowed by the interests of studying advanced parental age in modern society.^40^ Teenage parenthood is often associated with a less supportive and less stable home environment and impaired socioeconomic and educational status that could affect pregnancy and child health.^41,42^ Industrialization and increasing educational attainment in developed countries have led to delayed reproduction among adults and a decreased incidence of teenage parenthood over the past decades.^43^ However, young parenthood was common in Denmark during the grandparental generations. Therefore, ASD risk in the grandchildren associated with younger and older grandparental age could be attributable to different social and biological mechanisms.^44,45^

### Strengths and Limitations

Our study has several strengths. First, we were able to construct nationwide population cohort samples in Denmark that spanned across 3 generations to evaluate the association between parental or grandparental age and risk of ASD in children. Information on sociodemographic characteristics of the parents and the grandparents was obtained from data routinely registered at birth in the DMBR and CRS, which eliminated recall bias. The latter is a major limitation for some previous multigenerational studies that have relied on self-reported grandparental exposures retrospectively. Ages of the mother and the father were both registered in the CRS, thus allowing comparisons of maternal and paternal origins of ASD risk. Furthermore, the sample size was sufficiently large to quantify risk associated with 6 or more categories of age classifications and to conduct coadjusted analyses of the spouse’s age. Diagnoses of ASD were ascertained from the Danish Psychiatric Central Register, which documents data with high validity.^46^ This outcome assessment approach also limited possible bias resulting from nonparticipation.

Several limitations of the present study should also be noted. First, our multigenerational cohort, following parents born from 1973 to 1990, did not have a complete follow-up of their reproductive age by the end of 2013 (ie, the follow-up is not sufficiently long to capture older parental age); therefore, bias associated with truncated data is likely to be introduced. We could not rule out that chance findings are possible, especially for the youngest and the oldest age categories with smaller sample sizes. In our analyses, by extending the study period of the multigenerational cohorts including parents born from 1942 to 1990 in Denmark, we observed some attenuations of the younger grandparental age effects, which could partially be explained by increased statistical precision with a large sample size. Results for advanced maternal and paternal grandfather age effect estimates were strengthened. Moreover, the grandparental age effects were largely unchanged in the model further accounting for parental age, suggesting that the observed grandparental age associations were independent of the potential parental age effects. Our findings should be replicated in future cohorts with complete distributions of prospective data from both grandparental age and parental age, which would allow an estimation of joint effect of both ages. Moreover, grandpaternal education was not available for adjustment; thus, residual confounding is possibly affecting our grandpaternal age analyses. In addition, confounding by genetic risk for ASD might also be possible. The information on previous psychiatric diagnoses of the grandparents was also not available; thus, we were not able to adjust for these factors that might be associated with an individual’s chance of having a child or delaying their parenthood. Additional adjustment for psychiatric history of the parent, however, did not change the associations between parental age and ASD risk in children.

## Conclusions

These findings suggest that parental age of 30 years or greater is associated with an increased risk of ASD in the offspring. Younger or older age of grandparents at the time of the parent’s birth were also associated with ASD risk in grandchildren. Our findings enrich the current understanding of the complex causes of ASD. Our findings also highlight the potential adverse association of very young age at pregnancy and neurodevelopmental risk that has often been overlooked, particularly for the risk for ASD. Further studies on the role of transgenerational age effects may be important in understanding the role of reproductive age–related mechanisms in ASD and other related neurodevelopmental disorders.

## Notes

Supplement. **eTable 1. **Odds Ratios and 95% Confidence Intervals for Diagnosis of ASD in Children According to Continuous Parental Age (per 5-Year Increase) at Delivery **eTable 2.** Odds Ratios and 95% Confidence Intervals for Diagnosis of ASD in Children According to Parental Age (Years) at Delivery (Restricted to Children Born 2000-2013) **eTable 3.** Odds Ratios and 95% Confidence Intervals for Diagnosis of ASD in Children According to Paternal Age (Years) at Delivery, Further Adjustment of Paternal Education **eTable 4.** Effect Estimate for Diagnosis of ASD in Children According to Continuous Grandparental Age (per 5-Year Increase) at Delivery **eTable 5.** Odds Ratios and 95% Confidence Intervals for Diagnosis of ASD in Children According to Parental and Grandparental Age (years) at Delivery (Stratified by ASD Types) **eTable 6.** Odds Ratios and 95% Confidence Intervals for Diagnosis of ASD in Children According to Parental and Grandparental Age (years) at Delivery (Stratified by Child’s Sex) **eTable 7.** Odds Ratios and 95% Confidence Intervals for Diagnosis of ASD in Children According to Parental and Grandparental Age (Years) at Delivery (Stratified By Maternal or Grandmaternal Parity) **eTable 8.** Odds Ratios and 95% Confidence Intervals for Diagnosis of ASD in Grandchildren According to Grandparental Age (Years) at Delivery (Extent Multigenerational Cohort to Parents Born 1942-1990 Relinked From CRS) **eFigure 1.** The Directed Acyclic Graph (DAG) for the Main Variables Included in the Analyses for Parental Age and ASD Risk in Children **eFigure 2. **The Directed Acyclic Graph (DAG) for the Main Variables Included in the Analyses for Grandparental Age and ASD Risk in Grandchildren **eFigure 3.** Mean Age at Birth of the Parents and Grandparents in Parental Age Cohort and Multigenerational Cohort During the Study Period

## References

[1] American Psychiatric Association Diagnostic and Statistical Manual of Mental Disorders. 4th ed American Psychiatric Association; 1994.

[2] Christensen DL, Braun KVN, Baio J, Prevalence and characteristics of autism spectrum disorder among children aged 8 years: Autism and Developmental Disabilities Monitoring Network, 11 sites, United States, 2012. MMWR Surveill Summ. 2018;65(13):-. doi:10.15585/mmwr.ss6513a1 30439868 PMC6237390

[3] Baio J, Wiggins L, Christensen DL, Prevalence of autism spectrum disorder among children aged 8 years: Autism and Developmental Disabilities Monitoring Network, 11 sites, United States, 2014. MMWR Surveill Summ. 2018;67(6):1-23. doi:10.15585/mmwr.ss6706a1 29701730 PMC5919599

[4] Hansen SN, Schendel DE, Parner ET Explaining the increase in the prevalence of autism spectrum disorders: the proportion attributable to changes in reporting practices. JAMA Pediatr. 2015;169(1):56-62. doi:10.1001/jamapediatrics.2014.1893 25365033

[5] Lyall K, Croen L, Daniels J, The changing epidemiology of autism spectrum disorders. Annu Rev Public Health. 2017;38:81-102. doi:10.1146/annurev-publhealth-031816-044318 28068486 PMC6566093

[6] Kim JY, Son MJ, Son CY, Environmental risk factors and biomarkers for autism spectrum disorder: an umbrella review of the evidence. Lancet Psychiatry. 2019;6(7):590-600. doi:10.1016/S2215-0366(19)30181-6 31230684

[7] Myrskylä M, Silventoinen K, Tynelius P, Rasmussen F Is later better or worse? association of advanced parental age with offspring cognitive ability among half a million young Swedish men. Am J Epidemiol. 2013;177(7):649-655. doi:10.1093/aje/kws237 23467498

[8] Durkin MS, Maenner MJ, Newschaffer CJ, Advanced parental age and the risk of autism spectrum disorder. Am J Epidemiol. 2008;168(11):1268-1276. doi:10.1093/aje/kwn250 18945690 PMC2638544

[9] Grether JK, Anderson MC, Croen LA, Smith D, Windham GC Risk of autism and increasing maternal and paternal age in a large north American population. Am J Epidemiol. 2009;170(9):1118-1126. doi:10.1093/aje/kwp247 19783586

[10] Lampi KM, Hinkka-Yli-Salomäki S, Lehti V, Parental age and risk of autism spectrum disorders in a Finnish national birth cohort. J Autism Dev Disord. 2013;43(11):2526-2535. doi:10.1007/s10803-013-1801-3 23479075

[11] Parner ET, Baron-Cohen S, Lauritsen MB, Parental age and autism spectrum disorders. Ann Epidemiol. 2012;22(3):143-150. doi:10.1016/j.annepidem.2011.12.006 22277122 PMC4562461

[12] Wu S, Wu F, Ding Y, Hou J, Bi J, Zhang Z Advanced parental age and autism risk in children: a systematic review and meta-analysis. Acta Psychiatr Scand. 2017;135(1):29-41. doi:10.1111/acps.12666 27858958

[13] Iossifov I, Ronemus M, Levy D, De novo gene disruptions in children on the autistic spectrum. Neuron. 2012;74(2):285-299. doi:10.1016/j.neuron.2012.04.009 22542183 PMC3619976

[14] Kong A, Frigge ML, Masson G, Rate of de novo mutations and the importance of father’s age to disease risk. Nature. 2012;488(7412):471-475. doi:10.1038/nature11396 22914163 PMC3548427

[15] Sandin S, Hultman CM, Kolevzon A, Gross R, MacCabe JH, Reichenberg A Advancing maternal age is associated with increasing risk for autism: a review and meta-analysis. J Am Acad Child Adolesc Psychiatry. 2012;51(5):477.e1-486.e1. doi:10.1016/j.jaac.2012.02.018 22525954

[16] McCarthy DM, Morgan TJ Jr, Lowe SE, Nicotine exposure of male mice produces behavioral impairment in multiple generations of descendants. PLoS Biol. 2018;16(10):e2006497. doi:10.1371/journal.pbio.2006497 30325916 PMC6191076

[17] Sampino S, Juszczak GR, Zacchini F, Grand-paternal age and the development of autism-like symptoms in mice progeny. Transl Psychiatry. 2014;4:e386. doi:10.1038/tp.2014.27 24780920 PMC4012289

[18] Choi CS, Gonzales EL, Kim KC, The transgenerational inheritance of autism-like phenotypes in mice exposed to valproic acid during pregnancy. Sci Rep. 2016;6:36250. doi:10.1038/srep36250 27819277 PMC5098241

[19] Golding J, Ellis G, Gregory S, Grand-maternal smoking in pregnancy and grandchild’s autistic traits and diagnosed autism. Sci Rep. 2017;7:46179. doi:10.1038/srep46179 28448061 PMC5407180

[20] Kioumourtzoglou MA, Coull BA, O’Reilly EJ, Ascherio A, Weisskopf MG Association of exposure to diethylstilbestrol during pregnancy with multigenerational neurodevelopmental deficits. JAMA Pediatr. 2018;172(7):670-677. doi:10.1001/jamapediatrics.2018.0727 29799929 PMC6137513

[21] Frans EM, Sandin S, Reichenberg A, Autism risk across generations: a population-based study of advancing grandpaternal and paternal age. JAMA Psychiatry. 2013;70(5):516-521. doi:10.1001/jamapsychiatry.2013.1180 23553111 PMC3701020

[22] Frans EM, McGrath JJ, Sandin S, Advanced paternal and grandpaternal age and schizophrenia: a three-generation perspective. Schizophr Res. 2011;133(1-3):120-124. doi:10.1016/j.schres.2011.09.027 22000939 PMC3660090

[23] Schendel DE, Overgaard M, Christensen J, Association of psychiatric and neurologic comorbidity with mortality among persons with autism spectrum disorder in a Danish population. JAMA Pediatr. 2016;170(3):243-250. doi:10.1001/jamapediatrics.2015.3935 26752506

[24] Bliddal M, Broe A, Pottegård A, Olsen J, Langhoff-Roos J The Danish medical birth register. Eur J Epidemiol. 2018;33(1):27-36. doi:10.1007/s10654-018-0356-1 29349587

[25] Munk-Jørgensen P, Kastrup M, Mortensen PB The Danish psychiatric register as a tool in epidemiology. Acta Psychiatr Scand Suppl. 1993;370:27-32. doi:10.1111/j.1600-0447.1993.tb05358.x 8452052

[26] Nissen J, Powell S, Koch SV, Diagnostic validity of early-onset obsessive-compulsive disorder in the Danish Psychiatric Central Register: findings from a cohort sample. BMJ Open. 2017;7(9):e017172. doi:10.1136/bmjopen-2017-017172 28928194 PMC5623479

[27] Maenner MJ, Schieve LA, Rice CE, Frequency and pattern of documented diagnostic features and the age of autism identification. J Am Acad Child Adolesc Psychiatry. 2013;52(4):401-413. doi:10.1016/j.jaac.2013.01.014 23582871 PMC4051284

[28] Li J, Vestergaard M, Obel C, Cnattingus S, Gissler M, Olsen J Cohort profile: the Nordic Perinatal Bereavement Cohort. Int J Epidemiol. 2011;40(5):1161-1167. doi:10.1093/ije/dyq127 20675718

[29] Lee BK, McGrath JJ Advancing parental age and autism: multifactorial pathways. Trends Mol Med. 2015;21(2):118-125. doi:10.1016/j.molmed.2014.11.005 25662027

[30] Sandin S, Schendel D, Magnusson P, Autism risk associated with parental age and with increasing difference in age between the parents. Mol Psychiatry. 2016;21(5):693-700. doi:10.1038/mp.2015.70 26055426 PMC5414073

[31] Idring S, Magnusson C, Lundberg M, Parental age and the risk of autism spectrum disorders: findings from a Swedish population-based cohort. Int J Epidemiol. 2014;43(1):107-115. doi:10.1093/ije/dyt262 24408971

[32] Croen LA, Najjar DV, Fireman B, Grether JK Maternal and paternal age and risk of autism spectrum disorders. Arch Pediatr Adolesc Med. 2007;161(4):334-340. doi:10.1001/archpedi.161.4.334 17404129

[33] Golding J, Steer C, Pembrey M Parental and grandparental ages in the autistic spectrum disorders: a birth cohort study. PLoS One. 2010;5(4):e9939. doi:10.1371/journal.pone.0009939 20376340 PMC2848579

[34] Girard SL, Bourassa CV, Lemieux Perreault LP, Paternal age explains a major portion of de novo germline mutation rate variability in healthy individuals. PLoS One. 2016;11(10):e0164212. doi:10.1371/journal.pone.0164212 27723766 PMC5056704

[35] Gratten J, Wray NR, Peyrot WJ, McGrath JJ, Visscher PM, Goddard ME Risk of psychiatric illness from advanced paternal age is not predominantly from de novo mutations. Nat Genet. 2016;48(7):718-724. doi:10.1038/ng.3577 27213288

[36] Ni G, Gratten J, Wray NR, Lee SH; Schizophrenia Working Group of the Psychiatric Genomics Consortium Age at first birth in women is genetically associated with increased risk of schizophrenia. Sci Rep. 2018;8(1):10168. doi:10.1038/s41598-018-28160-z 29977057 PMC6033923

[37] Mehta D, Tropf FC, Gratten J, ; Schizophrenia Working Group of the Psychiatric Genomics Consortium, LifeLines Cohort Study, and TwinsUK Evidence for genetic overlap between schizophrenia and age at first birth in women. JAMA Psychiatry. 2016;73(5):497-505. doi:10.1001/jamapsychiatry.2016.0129 27007234 PMC5785705

[38] Sharma R, Agarwal A, Rohra VK, Assidi M, Abu-Elmagd M, Turki RF Effects of increased paternal age on sperm quality, reproductive outcome and associated epigenetic risks to offspring. Reprod Biol Endocrinol. 2015;13:35. doi:10.1186/s12958-015-0028-x 25928123 PMC4455614

[39] Puleo CM, Reichenberg A, Smith CJ, Kryzak LA, Silverman JM Do autism-related personality traits explain higher paternal age in autism? Mol Psychiatry. 2008;13(3):243-244. doi:10.1038/sj.mp.4002102 18285759

[40] McGrath JJ, Petersen L, Agerbo E, Mors O, Mortensen PB, Pedersen CB A comprehensive assessment of parental age and psychiatric disorders. JAMA Psychiatry. 2014;71(3):301-309. doi:10.1001/jamapsychiatry.2013.4081 24452535

[41] Alio AP, Mbah AK, Grunsten RA, Salihu HM Teenage pregnancy and the influence of paternal involvement on fetal outcomes. J Pediatr Adolesc Gynecol. 2011;24(6):404-409. doi:10.1016/j.jpag.2011.07.002 22099734

[42] Boden JM, Fergusson DM, John Horwood L Early motherhood and subsequent life outcomes. J Child Psychol Psychiatry. 2008;49(2):151-160. doi:10.1111/j.1469-7610.2007.01830.x 18093114

[43] Huber S, Bookstein FL, Fieder M Socioeconomic status, education, and reproduction in modern women: an evolutionary perspective. Am J Hum Biol. 2010;22(5):578-587. doi:10.1002/ajhb.21048 20737603

[44] Durkin MS, Maenner MJ, Meaney FJ, Socioeconomic inequality in the prevalence of autism spectrum disorder: evidence from a U.S. cross-sectional study. PLoS One. 2010;5(7):e11551. doi:10.1371/journal.pone.0011551 20634960 PMC2902521

[45] Yeshurun S, Hannan AJ Transgenerational epigenetic influences of paternal environmental exposures on brain function and predisposition to psychiatric disorders. Mol Psychiatry. 2019;24(4):536-548. doi:10.1038/s41380-018-0039-z 29520039

[46] Lauritsen MB, Jørgensen M, Madsen KM, Validity of childhood autism in the Danish Psychiatric Central Register: findings from a cohort sample born 1990-1999. J Autism Dev Disord. 2010;40(2):139-148. doi:10.1007/s10803-009-0818-0 19728067

