# Supplementary material for: Association of Grandparental and Parental Age at Childbirth With Autism Spectrum Disorder in Children
Source: JAMA Netw Open. 2020 Apr 15;3(4):e202868. doi: 10.1001/jamanetworkopen.2020.2868 (PMC7160691; doi:10.1001/jamanetworkopen.2020.2868)
Supplement: Supplement. — eTable 1. Odds Ratios and 95% Confidence Intervals for Diagnosis of ASD in Children According to Continuous Parental Age (per 5-Year Increase) at Delivery eTable 2. Odds Ratios and 95% Confidence Intervals for Diagnosis of ASD in Children According to Parental Age (Years) at Delivery (Restricted to Children Born 2000-2013) eTable 3. Odds Ratios and 95% Confidence Intervals for Diagnosis of ASD in Children According to Paternal Age (Years) at Delivery, Further Adjustment of Paternal Education eTable 4. Effect Estimate for Diagnosis of ASD in Children According to Continuous Grandparental Age (per 5-Year Increase) at Delivery eTable 5. Odds Ratios and 95% Confidence Intervals for Diagnosis of ASD in Children According to Parental and Grandparental Age (years) at Delivery (Stratified by ASD Types) eTable 6. Odds Ratios and 95% Confidence Intervals for Diagnosis of ASD in Children According to Parental and Grandparental Age (years) at Delivery (Stratified by Child’s Sex) eTable 7. Odds Ratios and 95% Confidence Intervals for Diagnosis of ASD in Children According to Parental and Grandparental Age (Years) at Delivery (Stratified By Maternal or Grandmaternal Parity) eTable 8. Odds Ratios and 95% Confidence Intervals for Diagnosis of ASD in Grandchildren According to Grandparental Age (Years) at Delivery (Extent Multigenerational Cohort to Parents Born 1942-1990 Relinked From CRS) eFigure 1. The Directed Acyclic Graph (DAG) for the Main Variables Included in the Analyses for Parental Age and ASD Risk in Children eFigure 2. The Directed Acyclic Graph (DAG) for the Main Variables Included in the Analyses for Grandparental Age and ASD Risk in Grandchildren eFigure 3. Mean Age at Birth of the Parents and Grandparents in Parental Age Cohort and Multigenerational Cohort During the Study Period [file jamanetwopen-3-e202868-s001.pdf]

Supplementary Online Content

Gao Y, Yu Y, Xiao J, et al. Association of grandparental and parental age at childbirth with autism spectrum disorder in children. *JAMA Netw Open*. 2020;3(4):e202868. doi:10.1001/jamanetworkopen.2020.2868

eTable 1. Odds Ratios and 95% Confidence Intervals for Diagnosis of ASD in Children According to Continuous Parental Age (per 5-Year Increase) at Delivery

eTable 2. Odds Ratios and 95% Confidence Intervals for Diagnosis of ASD in Children According to Parental Age (Years) at Delivery (Restricted to Children Born 2000-2013)

eTable 3. Odds Ratios and 95% Confidence Intervals for Diagnosis of ASD in Children According to Paternal Age (Years) at Delivery, Further Adjustment of Paternal Education

eTable 4. Effect Estimate for Diagnosis of ASD in Children According to Continuous Grandparental Age (per 5-Year Increase) at Delivery

eTable 5. Odds Ratios and 95% Confidence Intervals for Diagnosis of ASD in Children According to Parental and Grandparental Age (years) at Delivery (Stratified by ASD Types)

eTable 6. Odds Ratios and 95% Confidence Intervals for Diagnosis of ASD in Children According to Parental and Grandparental Age (years) at Delivery (Stratified by Child's Sex)

eTable 7. Odds Ratios and 95% Confidence Intervals for Diagnosis of ASD in Children According to Parental and Grandparental Age (Years) at Delivery (Stratified By Maternal or Grandmaternal Parity)

eTable 8. Odds Ratios and 95% Confidence Intervals for Diagnosis of ASD in Grandchildren According to Grandparental Age (Years) at Delivery (Extent Multigenerational Cohort to Parents Born 1942-1990 Relinked From CRS)

eFigure 1. The Directed Acyclic Graph (DAG) for the Main Variables Included in the Analyses for Parental Age and ASD Risk in Children

eFigure 2. The Directed Acyclic Graph (DAG) for the Main Variables Included in the Analyses for Grandparental Age and ASD Risk in Grandchildren

eFigure 3. Mean Age at Birth of the Parents and Grandparents in Parental Age Cohort and Multigenerational Cohort During the Study Period

This supplementary material has been provided by the authors to give readers additional information about their work.

© 2020 Gao Y et al. *JAMA Network Open.*

eTable 1. Odds Ratios and 95% Confidence Intervals for Diagnosis of ASD in Children According to Continuous Parental Age (per 5-Year Increase) at Delivery

	Model 1		Model 2	
	OR	95% CI	OR	95% CI
Maternal age[a]	1.09	1.07-1.10	1.03	1.02-1.05
Paternal age[a]	1.09	1.08-1.10	1.07	1.06-1.09

OR: Odds ratios 95% CI: 95% Confidence Intervals
Continuous age variable was re-centered to age 25 and rescaled for per 5-year increase.
Model 1: Adjust for child birth year, maternal parity, maternal education, maternal country of origin.
Model 2: Adjust for child birth year, maternal parity, maternal education, maternal country of origin, spouse age.

© 2020 Gao Y et al. *JAMA Network Open.*

eTable 2. Odds Ratios and 95% Confidence Intervals for Diagnosis of ASD in Children According to Parental Age (Years) at Delivery (Restricted to Children Born 2010-2013)

	ASD		Model 1		Model 2		Model 3	
Maternal age, years	N of cases	N of non-cases	OR	95% CI	OR	95% CI	OR	95% CI
≤19	202	8688	0.92	0.80-1.07	0.91	0.77-1.07	0.88	0.76-1.02
20-24	1728	82187	0.99	0.93-1.05	0.99	0.93-1.05	0.96	0.90-1.02
25-29	4710	257672	reference		reference		reference	
30-34	5181	308896	1.06	1.02-1.11	1.04	0.99-1.09	1.08	1.04-1.13
35-39	2402	143440	1.17	1.11-1.23	1.09	1.03-1.15	1.21	1.15-1.27
≥40	586	26982	1.59	1.46-1.74	1.40	1.27-1.54	1.66	1.52-1.82
Paternal age, years								
≤19	60	2427	0.99	0.76-1.29	1.05	0.79-1.38	0.96	0.74-1.25
20-24	857	39287	1.02	0.95-1.11	1.03	0.95-1.13	1.00	0.92-1.08
25-29	3363	178463	reference		reference		reference	
30-34	5190	303245	1.04	0.99-1.08	1.02	0.97-1.07	1.05	1.00-1.10
35-39	3383	201894	1.11	1.06-1.17	1.07	1.01-1.13	1.14	1.08-1.20
40-44	1393	74378	1.31	1.23-1.40	1.21	1.13-1.30	1.36	1.27-1.45
45-49	395	20911	1.36	1.22-1.51	1.21	1.08-1.36	1.40	1.26-1.56
≥50	168	7260	1.64	1.40-1.92	1.46	1.24-1.71	1.69	1.46-1.98

Restricted children born 2000-2013

OR: Odds ratios 95% CI: 95% Confidence Intervals

Model 1: Adjust for child birth year, maternal parity, maternal education, maternal country of origin

Model 2: Adjust for child birth year, maternal parity, maternal education, maternal country of origin, spouse age

Model 3: Adjust for child birth year, maternal parity, maternal education, maternal country of origin, family psychiatric history

© 2020 Gao Y et al. *JAMA Network Open.*

eTable 3. Odds Ratios and 95% Confidence Intervals for Diagnosis of ASD in Children According to Paternal Age (Years) at Delivery, Further Adjustment of Paternal Education

	ASD		Model 1		Model 2	
	No of cases	No of Non-cases	OR	95% CI	OR	95% CI
Paternal age						
≤19	120	5093	0.92	0.76-1.12	0.97	0.79-1.19
20-24	1886	85626	1.00	0.95-1.05	1.00	0.94-1.05
25-29	7028	362535	reference		reference	
30-34	9464	526094	1.03	1.00-1.06	1.02	0.99-1.06
35-39	5838	316467	1.15	1.10-1.19	1.11	1.07-1.16
40-44	2322	111036	1.36	1.29-1.43	1.27	1.20-1.34
45-49	693	31262	1.45	1.34-1.58	1.33	1.22-1.45
≥50	265	11054	1.53	1.35-1.74	1.40	1.23-1.60

Model 1: Adjust for child birth year, maternal parity, , maternal education, maternal country of origin.

Model 2: Adjust for child birth year, maternal parity, maternal education, maternal country of origin, paternal education.

Information of paternal education was obtained from the Integrated Database for Labour Market Research. About 5% (1381cases and 72458 non-cases) without paternal education information were excluded in Model 2.

© 2020 Gao Y et al. *JAMA Network Open.*

eTable 4. Odds Ratios and 95% Confidence Intervals for Diagnosis of ASD in Children According to Continuous Grandparental Age (per 5-Year Increase) at Delivery

		Linear age term		Squared age term		
Model 1	intercept	β	SE	β	SE	*P* value
Maternal grandmother age[a]	-3.98	-0.87	0.11	0.066	0.010	<0.01
Maternal grandfather age[a]	-3.92	-0.43	0.08	0.029	0.006	<0.01
Paternal grandmother age[b]	-4.17	-0.39	0.13	0.032	0.012	0.008
Paternal grandfather age[b]	-4.15	-0.30	0.09	0.023	0.007	0.001
Model 2						
Maternal grandmother age[c]	-3.97	-0.79	0.12	0.061	0.011	<0.001
Maternal grandfather age[c]	-3.97	-0.10	0.09	0.007	0.007	0.308
Paternal grandmother age[d]	-4.19	-0.24	0.16	0.018	0.014	0.198
Paternal grandfather age[d]	-4.19	-0.35	0.20	0.031	0.016	0.056

OR: Odds ratios 95% CI: 95% Confidence Intervals

Continuous age variable was re-centered to age 25 and rescaled for per 5-year increase. A linear term and a squared term of age were included in the logistic regression models.

Model 1: a: Adjust for maternal birth year, maternal grandmaternal parity, maternal grandmother education.

b: Adjust for paternal birth year, paternal grandmaternal parity, paternal grandmother education.

Model 2: c: Adjust for maternal birth year, maternal grandmaternal parity, maternal grandmother education, maternal grand spouse age

d: Adjust for paternal birth year, paternal grandmaternal parity, paternal grandmother education, paternal grand spouse age

© 2020 Gao Y et al. *JAMA Network Open.*

eTable 5. Odds Ratios and 95% Confidence Intervals for Diagnosis of ASD in Children According to Parental and Grandparental Age (Years) at Delivery (Stratified by ASD Types)

	Childhood autism (F84.0)				Asperger's syndrome (F84.5)				Other types			
Maternal age, years[a]	N of cases	N of non-cases	OR	95% CI	N of cases	N of non-cases	OR	95% CI	N of cases	N of non-cases	OR	95% CI
≤19	129	19005	0.84	0.70-1.01	84	19005	0.75	0.60-0.94	258	19005	1.06	0.93-1.21
20-24	1152	176842	0.97	0.90-1.04	1025	176842	1.02	0.94-1.10	2266	176842	1.12	1.06-1.18
25-29	2769	498454	reference		2731	498454	reference		5100	498454	reference	
30-34	2803	507045	1.08	1.02-1.14	2572	507045	1.12	1.06-1.18	5111	507045	1.08	1.03-1.12
35-39	1352	211177	1.30	1.21-1.39	1054	211177	1.31	1.22-1.41	2166	211177	1.17	1.11-1.24
≥40	293	36644	1.61	1.42-1.82	206	36644	1.70	1.47-1.97	475	36644	1.54	1.40-1.70
Paternal age, years[a]												
≤19	32	5093	0.79	0.56-1.12	24	5093	0.77	0.52-1.16	78	5093	1.10	0.87-1.38
20-24	529	85626	0.93	0.84-1.02	525	85626	1.06	0.96-1.17	1063	85626	1.02	0.96-1.10
25-29	2044	362535	reference		2051	362535	reference		3952	362535	reference	
30-34	2844	526094	1.03	0.97-1.10	2711	526094	1.06	1.00-1.12	5216	526094	0.99	0.95-1.03
35-39	1857	316467	1.17	1.09-1.25	1540	316467	1.17	1.09-1.25	3282	316467	1.11	1.06-1.17
40-44	844	111036	1.50	1.38-1.63	575	111036	1.37	1.25-1.51	1266	111036	1.27	1.19-1.35
45-49	235	31262	1.47	1.28-1.69	179	31262	1.57	1.34-1.83	386	31262	1.41	1.26-1.56
≥50	113	11054	1.92	1.59-2.33	67	11054	1.69	1.32-2.16	133	11054	1.37	1.15-1.64
Maternal grandmother age[b]												
≤19	234	23418	1.90	1.62-2.23	118	23418	1.85	1.48-2.32	338	23418	1.53	1.34-1.74
20-24	1044	157907	1.26	1.15-1.39	564	157907	1.34	1.17-1.53	1785	157907	1.23	1.15-1.33
25-29	942	176418	reference		496	176418	reference		1620	176418	reference	
30-34	454	72521	1.13	1.00-1.27	204	72521	0.95	0.80-1.13	680	72521	0.99	0.90-1.09
≥35	134	20233	1.15	0.95-1.39	56	20233	0.90	0.68-1.19	197	20233	0.99	0.85-1.16
Maternal grandfather age[b]												
≤19	52	5578	1.59	1.19-2.11	35	5578	2.01	1.42-2.85	79	5578	1.39	1.10-1.75
20-24	646	88583	1.26	1.14-1.40	325	88583	1.17	1.02-1.35	1097	88583	1.24	1.14-1.34
25-29	1059	186817	reference		580	186817	reference		1841	186817	reference	

© 2020 Gao Y et al. *JAMA Network Open.*

30-34	662	114277	1.02	0.92-1.13	344	114277	0.98	0.85-1.13	1061	114277	0.94	0.86-1.01
35-39	282	38889	1.25	1.08-1.43	113	38889	0.92	0.75-1.14	368	38889	0.93	0.82-1.04
≥40	107	16353	1.10	0.89-1.35	41	16353	0.74	0.53-1.02	176	16353	1.01	0.86-1.19
Paternal grandmother age[c]												
≤19	131	17246	1.23	1.00-1.50	45	17246	1.09	0.77-1.52	191	17246	1.16	0.98-1.36
20-24	757	121846	1.06	0.95-1.18	309	121846	1.06	0.90-1.25	1245	121846	1.12	1.03-1.22
25-29	800	142482	reference		349	142482	reference		1261	142482	reference	
30-34	337	59116	1.03	0.90-1.17	143	59116	0.99	0.81-1.21	534	59116	1.01	0.91-1.12
≥35	97	16173	1.06	0.85-1.32	35	16173	0.87	0.61-1.25	163	16173	1.08	0.91-1.28
Paternal grandfather age[c]												
≤24	481	72627	1.11	0.99-1.25	187	72627	1.07	0.89-1.29	764	72627	1.12	1.02-1.23
25-29	853	149105	reference		368	149105	reference		1358	149105	reference	
30-34	521	91618	1.02	0.91-1.14	211	91618	0.95	0.80-1.13	858	91618	1.03	0.95-1.13
35-39	185	30769	1.07	0.91-1.27	77	30769	1.03	0.80-1.34	270	30769	0.95	0.83-1.09
≥40	82	12744	1.11	0.88-1.41	38	12744	1.17	0.83-1.65	144	12744	1.16	0.97-1.39

OR: Odds ratios 95% CI: 95% Confidence Intervals
a: Adjust for child birth year, maternal parity, maternal education, maternal country of origin
b: Adjust for maternal l birth year, maternal grandmaternal parity, maternal grandmother education
c: Adjust for paternal birth year, paternal granmaternal parity, paternal grandmother education

© 2020 Gao Y et al. *JAMA Network Open.*

eTable 6. Odds Ratios and 95% Confidence Intervals for Diagnosis of ASD in Children According to Parental and Grandparental Age (Years) at Delivery (Stratified by Child's Sex)

	ASD of Male				ASD of Female			
Maternal age[a]	N of cases	N of non-cases	OR	95% CI	N of cases	N of non-cases	OR	95% CI
≤19	334	9691	0.97	0.87-1.09	85	9312	0.76	0.61-0.95
20-24	2928	89833	1.05	1.00-1.10	965	86989	1.02	0.94-1.10
25-29	6922	253502	reference		2431	244897	reference	
30-34	6725	258358	1.07	1.03-1.11	2430	248648	1.10	1.03-1.16
35-39	2948	107585	1.22	1.16-1.28	1012	103584	1.20	1.11-1.30
≥40	610	18630	1.53	1.40-1.66	226	18014	1.66	1.44-1.91
Paternal age[a]								
≤19	85	2564	0.90	0.72-1.12	35	2528	1.13	0.81-1.59
20-24	1429	43586	1.03	0.97-1.10	457	42030	0.98	0.89-1.09
25-29	5192	184514	reference		1836	177981	reference	
30-34	6962	268017	1.02	0.99-1.06	2502	258038	1.02	0.96-1.10
35-39	4349	161027	1.16	1.11-1.21	1489	155414	1.11	1.03-1.19
40-44	1738	56540	1.37	1.29-1.45	584	54489	1.31	1.19-1.44
45-49	516	15796	1.47	1.34-1.61	177	15466	1.44	1.23-1.69
≥50	196	5555	1.58	1.37-1.83	69	5498	1.58	1.24-2.02
Maternal grandmother age[b]								
≤19	473	11926	1.71	1.53-1.92	128	11490	1.54	1.25-1.90
20-24	2232	80240	1.24	1.16-1.32	725	77667	1.30	1.16-1.46
25-29	2031	89725	reference		650	86693	reference	
30-34	904	37093	1.03	0.95-1.12	264	35428	0.95	0.82-1.11
≥35	258	10332	1.01	0.89-1.15	72	9901	0.90	0.70-1.16
Maternal grandfather age[b]								
≤19	103	2822	1.43	1.16-1.75	38	2755	1.73	1.24-2.42
20-24	1377	45074	1.23	1.14-1.32	423	43508	1.21	1.07-1.37
25-29	2315	95266	reference		740	91551	reference	
30-34	1387	57881	0.98	0.92-1.05	426	56396	0.93	0.82-1.05
35-39	489	19871	0.98	0.88-1.08	165	19018	1.05	0.87-1.25
≥40	227	8402	1.02	0.89-1.18	47	7951	0.68	0.50-0.92
Paternal grandmother age[c]								
≤19	253	8959	1.19	1.03-1.39	75	8287	1.10	0.84-1.45
20-24	1555	62077	1.10	1.01-1.19	457	59769	1.06	0.90-1.23
25-29	1622	72627	reference		476	69855	reference	
30-34	672	30139	0.94	0.85-1.05	217	28977	1.11	0.91-1.34
≥35	186	8200	1.10	0.93-1.30	62	7973	1.19	0.88-1.62
Paternal grandfather age[c]								
≤24	971	36961	1.15	1.04-1.27	287	35666	1.13	0.95-1.36
25-29	1728	76054	reference		520	73051	reference	
30-34	1064	46758	1.03	0.94-1.13	321	44860	0.99	0.83-1.18
35-39	353	15819	0.98	0.85-1.13	106	14950	0.96	0.73-1.25
≥40	172	6410	1.12	0.92-1.36	53	6334	1.27	0.90-1.80

© 2020 Gao Y et al. *JAMA Network Open.*

OR: Odds ratios 95% CI: 95% Confidence Intervals
a: Adjust for child birth year, maternal parity, maternal education, maternal country of origin
b: Adjust for maternal l birth year, maternal grandmaternal parity, maternal grandmother education
c: Adjust for paternal birth year, paternal grandmaternal parity, paternal grandmother education

© 2020 Gao Y et al. *JAMA Network Open.*

eTable 7. Odds Ratios and 95% Confidence Intervals for Diagnosis of ASD in Children According to Parental and Grandparental Age (Years) at Delivery (Stratified by Maternal or Grandmaternal Parity)

	First-born children or parents[d]				All children or parents[e]			
Maternal age[a]	N of cases	N of non-cases	OR	95% CI	N of cases	N of non-cases	OR	95% CI
≤19	397	17416	0.95	0.86-1.06	419	19005	0.92	0.83-1.02
20-24	2922	126444	1.06	1.01-1.11	3893	176842	1.04	1.00-1.08
25-29	5436	271853	reference		9353	498454	Reference	
30-34	3633	165469	1.14	1.09-1.19	9155	507045	1.08	1.04-1.11
35-39	1117	45628	1.30	1.22-1.39	3960	211177	1.22	1.17-1.27
≥40	202	7284	1.53	1.33-1.76	836	36644	1.56	1.45-1.68
Paternal age[a]								
≤19	108	4688	0.94	0.77-1.14	120	5093	0.96	0.80-1.15
20-24	1463	65255	1.02	0.96-1.08	1886	85626	1.02	0.97-1.08
25-29	4480	220474	reference		7028	362535	Reference	
30-34	4432	213659	1.06	1.01-1.10	9464	526094	1.02	0.99-1.06
35-39	2096	87916	1.25	1.19-1.32	5838	316467	1.14	1.10-1.19
40-44	770	29010	1.43	1.32-1.54	2322	111036	1.35	1.29-1.42
45-49	254	9272	1.52	1.33-1.73	693	31262	1.46	1.35-1.58
≥50	104	3820	1.52	1.25-1.86	265	11054	1.57	1.39-1.78
Maternal grandmother age[b]								
≤19	545	20889	1.74	1.54-1.96	601	23418	1.68	1.52-1.85
20-24	1731	99029	1.25	1.14-1.36	2957	157907	1.25	1.18-1.33
25-29	744	55690	reference		2681	176418	reference	
30-34	135	9718	1.05	0.87-1.26	1168	72521	1.02	0.95-1.10
≥35	26	1917	0.98	0.66-1.46	330	20233	0.99	0.88-1.11
Maternal grandfather age[b]								
≤19	127	5070	1.46	1.21-1.75	141	5578	1.50	1.26-1.78
20-24	1252	64475	1.18	1.09-1.28	1800	88583	1.22	1.15-1.30
25-29	1296	83983	reference		3055	186817	reference	
30-34	374	25576	0.98	0.87-1.10	1813	114277	0.96	0.91-1.02
35-39	102	5595	1.21	0.99-1.48	654	38889	0.99	0.91-1.09
≥40	30	2544	0.76	0.53-1.10	274	16353	0.94	0.83-1.07
Paternal grandmother age[c]								
≤19	280	15303	1.12	0.96-1.30	328	17246	1.18	1.04-1.34
20-24	1174	76255	1.03	0.93-1.15	2012	121846	1.08	1.01-1.16
25-29	623	44165	reference		2098	142482	reference	
30-34	114	7738	1.10	0.89-1.35	889	59116	1.02	0.94-1.10
≥35	20	1462	0.89	0.55-1.42	248	16173	1.00	0.87-1.15
Paternal grandfather age[c]								
≤24	893	53448	1.11	0.99-1.24	1258	72627	1.11	1.03-1.19
25-29	934	65926	reference		2248	149105	reference	
30-34	287	19432	1.08	0.92-1.26	1385	91618	1.02	0.95-1.09
35-39	62	4304	1.10	0.81-1.48	459	30769	0.99	0.89-1.10
≥40	35	1813	1.14	0.73-1.79	225	12744	1.11	0.97-1.28

OR: Odds ratios 95% CI: 95% Confidence Intervals

a: Adjust for child birth year, maternal education, maternal country of origin

© 2020 Gao Y et al. *JAMA Network Open.*

b: Adjust for maternal birth year, maternal grandmother education
c: Adjust for paternal birth year, paternal grandmother education
d: First-born children in the parental age cohort analysis and first-born parents in the multigeneration cohort analyses
e: Same adjustment as model 1 in table 3

© 2020 Gao Y et al. *JAMA Network Open.*

eTable 8. Odds Ratios and 95% Confidence Intervals for Diagnosis of ASD in Grandchildren According to Grandparental Age (Years) at Delivery (Extent Multigenerational Cohort to Parents Born 1942-1990 Relinked From CRS)

Maternal grandmother age[a]	ASD		Model 1		Model 2		Model 3	
	N of cases	N of non-cases	OR	95% CI	OR	95% CI	OR	95% CI
≤19	1940	81537	1.26	1.20-1.33	1.27	1.19-1.34	1.20	1.14-1.26
20-24	9273	444091	1.11	1.08-1.15	1.12	1.08-1.15	1.09	1.06-1.13
25-29	8530	456385	reference		reference		reference	
30-34	4028	218897	0.99	0.95-1.03	0.97	0.93-1.01	0.99	0.96-1.03
35-39	1579	78827	1.07	1.02-1.13	0.99	0.93-1.05	1.07	1.01-1.13
≥40	396	20450	1.03	0.93-1.14	0.89	0.79-1.00	1.02	0.92-1.13
Maternal grandfather age[a]								
≤19	410	17776	1.16	1.05-1.28	1.01	0.91-1.13	1.12	1.01-1.24
20-24	5274	247965	1.07	1.03-1.11	1.00	0.96-1.03	1.05	1.01-1.08
25-29	9376	473698	reference		reference		reference	
30-34	5870	320830	0.93	0.90-0.97	0.98	0.95-1.02	0.94	0.91-0.97
35-39	2859	148249	0.99	0.95-1.03	1.05	1.00-1.11	0.99	0.95-1.03
40-44	1269	60593	1.06	1.00-1.13	1.15	1.07-1.23	1.06	1.00-1.12
45-49	483	21807	1.12	1.02-1.23	1.22	1.11-1.36	1.11	1.01-1.22
≥50	205	9269	1.12	0.97-1.28	1.22	1.06-1.42	1.10	0.96-1.27
Paternal grandmother age[b]								
≤19	1745	79200	1.15	1.09-1.21	1.12	1.05-1.19	1.12	1.06-1.18
20-24	8503	423714	1.06	1.02-1.09	1.05	1.02-1.09	1.04	1.01-1.08
25-29	8253	437424	reference		reference			
30-34	3940	214136	0.98	0.94-1.02	0.95	0.91-0.99	0.98	0.94-1.02
35-39	1599	80042	1.05	0.99-1.11	0.98	0.92-1.05	1.05	0.99-1.10
≥40	448	21182	1.10	1.00-1.21	1.00	0.89-1.11	1.09	0.99-1.20
Paternal grandfather age[b]								
≤19	404	17120	1.22	1.10-1.35	1.14	1.03-1.28	1.19	1.08-1.32
20-24	4930	238576	1.08	1.04-1.12	1.04	1.00-1.08	1.06	1.03-1.10
25-29	8525	449195	reference		reference		reference	
30-34	5809	309715	1.00	0.96-1.03	1.03	0.99-1.07	1.00	0.97-1.03
35-39	2879	148181	1.02	0.98-1.07	1.07	1.02-1.13	1.02	0.98-1.07
40-44	1276	62005	1.07	1.01-1.13	1.12	1.04-1.20	1.07	1.01-1.13
45-49	469	22046	1.10	1.00-1.21	1.15	1.03-1.27	1.10	1.00-1.21
≥50	196	8860	1.15	1.00-1.33	1.20	1.03-1.39	1.15	0.99-1.32

OR: Odds ratios 95% CI: 95% Confidence Intervals

a: Model 1: Adjust for maternal birth year
Model 2: Adjust for maternal birth year and spouse age
Model 3: Adjust for maternal birth year and maternal age

b: Model 1: Adjust for paternal birth year
Model 2: Adjust for paternal age and spouse age
Model 3: Adjust for paternal birth year and paternal age

© 2020 Gao Y et al. *JAMA Network Open.*

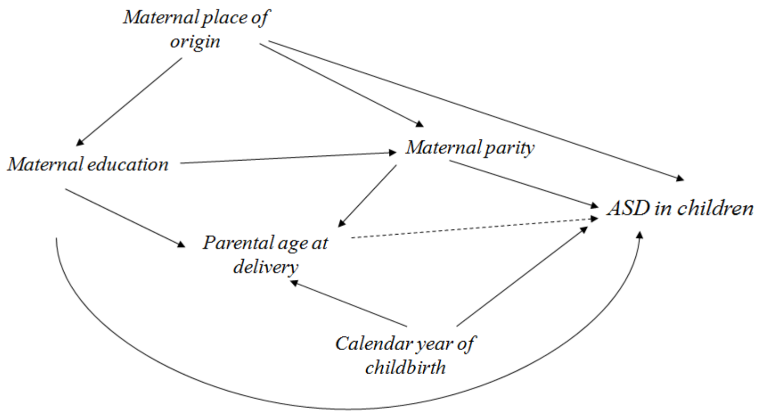

eFigure 1. The Directed Acyclic Graph (DAG) for the Main Variables Included in the Analyses for Parental Age and ASD Risk in Children

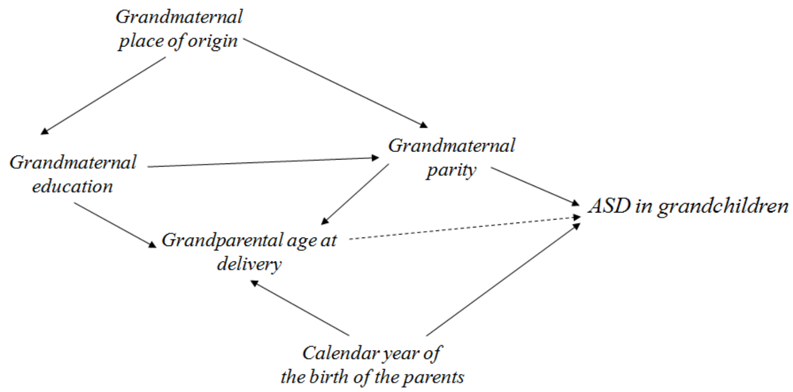

eFigure 2. The Directed Acyclic Graph (DAG) for the Main Variables Included in the Analyses for Grandparental Age and ASD Risk in Grandchildren

© 2020 Gao Y et al. *JAMA Network Open.*

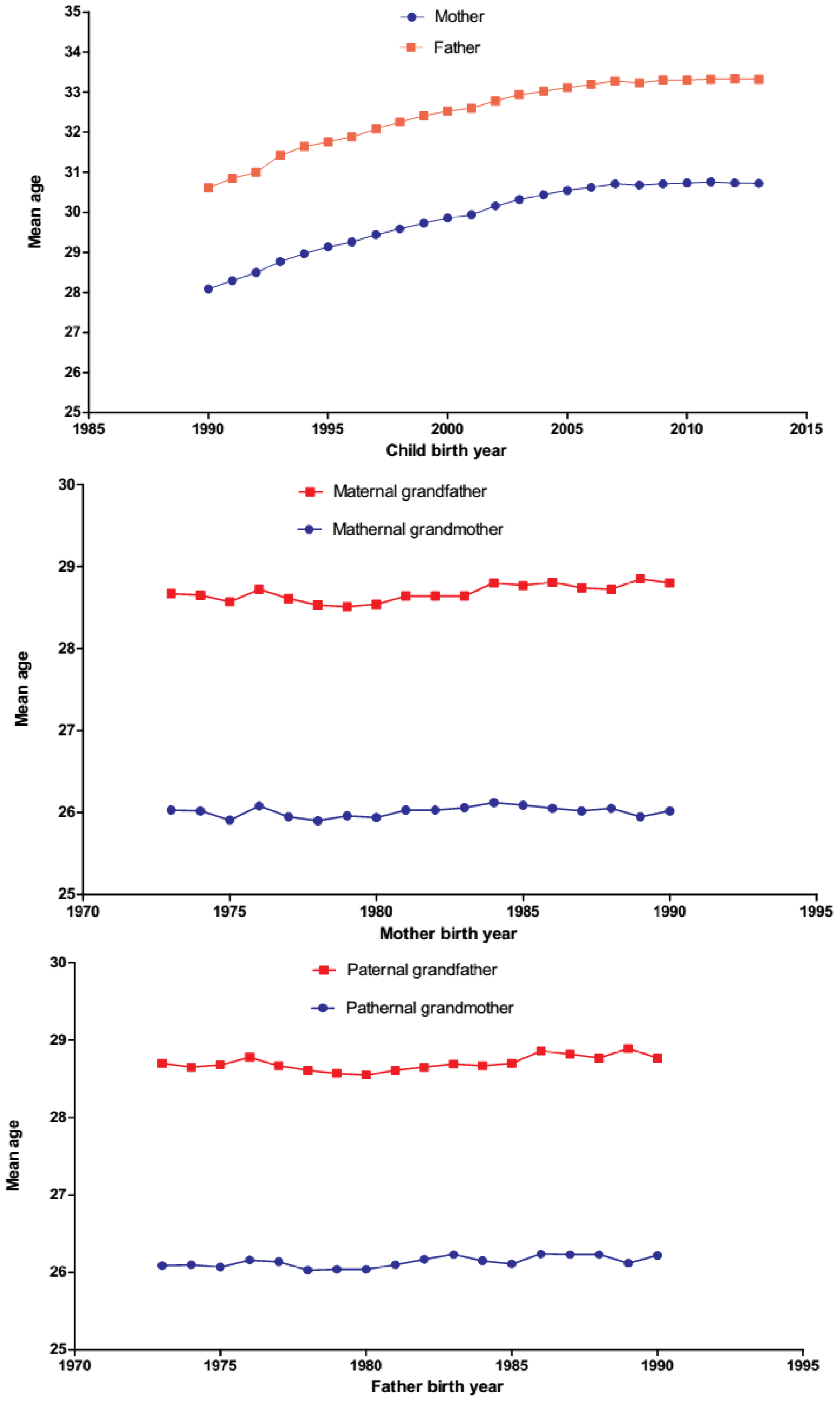

eFigure 3. Mean Age at Birth of the Parents and Grandparents in Parental Age Cohort and Multigenerational Cohort During the Study Period

© 2020 Gao Y et al. *JAMA Network Open.*
